# Stripe rust resistance gene(s) postulation in wheat germplasm with the help of differentials and tagged molecular markers

**DOI:** 10.1038/s41598-023-36197-y

**Published:** 2023-06-02

**Authors:** Mohammad Waris Haider, Jaspal Kaur, Ritu Bala, Sandeep Singh, Puja Srivastava, Achla Sharma, Rohtas Singh, Jyoti Kumari

**Affiliations:** 1 Department of Plant Pathology, PAU, Ludhiana, India; 2 Department of Plant Breeding and Genetics, PAU, Ludhiana, India; 3 National Bureau of Plant Genetic Resources, New Delhi, India

**Keywords:** Molecular biology, Plant sciences

## Abstract

Thirteen known *Yr* gene-associated markers pertaining to genes (*Yr5,* *Yr10,* *Yr15,* *Yr24/Yr26*) were used to identify the genes in selected wheat germplasm which were found resistant under field conditions at two locations in Punjab, India against stripe rust. In field evaluation, 38 genotypes exhibited highly resistant response, with a final rust severity (FRS) ranging from 0 to TR. Seven genotypes expressed a resistant to moderately resistant response with FRS ranging from 5MR–10S. In race-specific phenotying against most prevalent pathotypes of *Puccinia* *striiformis* *tritici* (46S119,110S119 &238S119) by seedling reaction test (SRT) 14 genotypes (29.2%) were found to be immune (IT = 0), 28 genotypes (58.3%) were resistant (IT = 1), and 3 genotypes (6.3%) were moderately resistant (IT = 2). *Yr5* was detected in sixteen lines with the help of two markers *Xwmc175* and *Xgwm120* linked with *Yr5*. *Yr10* was detected in ten lines with the marker *Xpsp3000* and *Yr15* was detected in fourteen lines with two linked markers; *Xgwm413* and *Xgwm273.* Likewise, *Yr24/26* was detected in 15 lines with two linked markers, namely *Xbarc181* and *Xbarc187.* Based on the race specific phenotyping data and marker data, fourteen lines were found to carry a single gene, 16 showed the presence of two gene combinations, and seven genotypes were found to have a combination of three genes. Frequencies of *Yr5*, *Yr15* and *Yr26/Yr24* was high among test wheat germplasm in comparison to *Yr10*.

## Introduction

Wheat, scientifically known as *Triticum* *aestivum* L., is the most widely consumed food grain, with a global per capita consumption of 67.4 kg/year^1^. In India, wheat production was recorded 106.41 MT in 2021–22, with 3484 kg/ha average national productivity^2^ Uttar Pradesh, Punjab, and Haryana are the main contributors to central pool in North-western Plains zone of India^3^. Wheat production in North-western Plains zone of India is threatened by a variety of factors, including pests and diseases. Approximately 16 percent losses due to pathogens have been reported globally^4,5^. Among-est the diseases stripe rust has been identified as the most devastating wheat disease, as it prefers a cool climate and can establish its infection during the early stages of crop growth and the infection prevails till the crop matures^6,7^. Under favourable conditions, this disease can result in yield losses of up to 90 percent^6,8^. In its moderate to a severe form, stripe rust caused 68.8% yield losses in Punjab during 2008^9^. *Puccinia* *striiformis*, the pathogen that causes stripe rust, is extremely diverse, possibly due to a combination of long-distance migration capacity^10^, high rates of mutation from avirulence to virulence^11^, adaptation to different climatic conditions^12^, the existence of recombinant and highly diverse populations^13,14^ and the potential creation of new variants through a sexual cycle^15^. Mutations in the pathogen's DNA can result in the emergence of new virulence races, allowing the pathogen to infect previously resistant plants^16,17^. In India, *P.* *striiformis* virulence in the cultivar Kalyansona was first appeared around 1971, followed by virulence in the cultivar Sonalika between 1984–1990^18^. The first detection of virulence for*Yr9* was in 1996, followed by a pathotype with combined virulence for *Yr9* and *Yr27* in 2001^19,20^. Finally, three new pathotypes 110S119, 238S119, and 110S84 with additional virulence have been identified on Riebesel 47/51, Suwon × Omar, *Yr11* and *Yr14*^21^. The resistance genes *Yr5,* *Yr10,* *Yr15* *Yr24/Yr26*, *Yr32* and *Yr*Sp are still effective against all the prevalent pathotypes of *P.* *striiformis* *tritici* in India, while *Yr2,* *Yr3,* *Yr4,* *Yr6,* *Yr7,* *Yr8,* *Yr9,* *Yr17,* *Yr18,* *Yr19,* *Yr21,* *Yr22,* *Yr23,* *Yr25,* *Yr27* and *YrA* have become ineffective to the prevailing and recently evolved pathotypes. Till date, 84 permanently designated stripe rust resistance genes, 100 temporarily designated genes, and 363 quantitative trait loci (QTLs) with different names have been reported in wheat^22–24^. Despite of so many genes have been identified which impart resistance against stripe rust throughout the crop season or at adult plant stage; we are left with only few effective *Yr* genes as many of them have been defeated due to the continuous and fast evolution of the pathogen in one or another part of the world. To broaden our gene pool for stripe rust resistance gene mining is a continuous process in the wheat breeding programs globally and further to achieve durability of resistance pyramiding is the need of the hour to keep pace with the ever-evolving pathogen. The known genes, namely *Yr5,* *Yr10,* *Yr15,* *Yr36,* *Yr40,* *Yr47* etc. have been extensively exploited in our in house breeding program at Punjab Agricultural University. However, the climate change and the fungal evolution is always anticipated to create newer pathotypes/races of the pathogen. In view of this, the novel genes need to be streamlined and characterized genetically for its ready mobilization whenever required. Since, the germplasm collections from National Bureau of Plant Genetic Resources (NBPGR) constitute the diverse germplasm collected from variable sites, it holds the promise to harbour newer and unidentified resistance sources. So, in the present study efforts were made to evaluate the wheat germplasm against the most prevalent and virulent pathotypes of the stripe rust pathogen At seedling stage as well as at adult plant response against stripe rust under field conditions and further molecular markers were deployed to identify the already known stripe rust resistance genes in the resistant germplasm.

## Materials and methods

### Plant materials

Total of 45 wheat accessions from NBPGR which showed highly resistant to resistant reaction in the preliminary screening of ~ 1500 wheat germplasm accessions from national gene bank of India, ICAR-National Bureau of Plant Genetic Resources (Table 1) were selected for the present study i.e. to identify the stripe rust resistance gene(s) present in them. This included 22 indigenous accessions collected or derived in India and 23 exotic collections augmented from USA, Mexico and Australia. These lines were evaluated against the three most prevalent races (238S119, 110S119, and 46S119) of stripe rust pathogen both at seedling and adult plant stages. The highly susceptible cultivars PBW343, HD2967, and Agra Local, as well as the Avocet background near-isogenic lines (NILs), i.e., Avocet/*Yr5*, Avocet/*Yr10*, Avocet/*Yr15*, Avocet/*Yr24*, and Avocet/*Yr26*, were used as susceptible and resistant checks respectively in the study for comparison purpose.

**Table 1:** List of wheat accessions used for gene postulation.

Sr no.	Accession	Alternate ID	Source/origin	Sr no.	Accession	Alternate ID	Source/origin	Sr no.	Accession	Alternate ID		Source/origin
1	IC111691	A-585	NBPGR, New Delhi	19	EC693322	37	Mexico	37	IC529094	VWFW-320		Almora, Uttarakhand
2	IC535470	NC-60205	NBPGR, New Delhi	20	EC692262	CW179116	Mexico	38	EC0582305	PI 520326		USA
3	IC415825	–	NBPGR, New Delhi	21	EC635577	–	Mexico	39	EC0612509	HSB3177		Australia
4	IC534662	PI322071	NBPGR, New Delhi	22	EC635750	–	Mexico	40	EC0612506	HSB2949		Australia
5	IC336645	22	Lahaul & Spiti,Himachal Pradesh	23	IC47034	428	Cuddapah,Andhra Pradesh	41	IC0591055	VL 900		Karnal, Haryana
6	EC217835	CITr 14174	USA	24	IC530087	VWFW-991	Almora, Uttarakhand	42	EC0610929	CS M1A		USA
7	EC339610	PI-519261 IPPO 9	USA	25	IC530078	VWFW-972	Almora, Uttarakhand	43	EC0612504	HSB2527		Australia
8	EC664198	487442	Mexico	26	IC443681	DBPY-2000–6	Karnal, Haryana	44	EC0105966	Goncha		Others
9	EC693621	–		27	IC445516	IDON CA04-115	Karnal, Haryana	45	IC0078981-B	BDJ-I-1046 (Kankoo)		Bilaspur, Himachal Pradesh
10	EC636264	–	Mexico	28	IC564090	KCM/PSM/RK-1051	Pauri Garhwal, Uttarakhand	46	*Avocet/Yr5*	+ ve control		
11	EC662236	–	NBPGR, New Delhi	29	IC553914	FLW-29	Shimla, Himachal Pradesh	47	*Avocet/Yr10*	+ ve control		
12	EC635701	–	Mexico	30	IC469420	VHC-6128	Chamoli, Uttarakhand	48	*Avocet/Yr15*	+ ve control		
13	EC0635709	–	Mexico	31	IC0591082	VL 925	Karnal, Haryana	49	*Avocet/Yr24*	+ ve control		
14	EC0610955	T2D.T44	USA	32	EC0635701		Mexico	51	*Avocet/Yr26*	+ ve control		
15	EC0635659	T2D.T44	USA	33	IC0078729-A		Uttar Pradesh	52	PBW343	Susceptible check		
16	IC0594933	–	Mexico	34	IC0078959-A	P-759	Uttar Pradesh	53	HD2967	Susceptible check		
17	ICO598571	SKS-49	Hisar,Haryana	35	ECO635685	K-3357-A	Mexico	54	Agra Local	Susceptible check		
18	EC0610944	CSM2D	USA	36	IC529052	–		55	A-9-30-1	Susceptible check		

### Seedling reaction test

Wheat seedlings were raised in plastic germination trays (14 × 7 cups) filled with a mixture of soil having sandy loam soil cocopeet + Farm Yard Mannure and vermi compost. All the germplasm were sown in three sets for evaluation against stripe rust pathotypes 46S119, 238S119, and 110S119 separately. The leaves of 10-day-old seedlings were inoculated with uredospores of three different pathotypes (46S119, 110S119 & 238S119) separately and kept in separate poly-chambers. For a successful infection, the inoculated material was placed in a dew chamber for 48 h in the dark before being transported to a greenhouse and maintained with a photo period of 16 h light and 8 h of darkness. Regular irrigation was given to maintain the humidity. The host response for seedling infection types was recorded 14 days after inoculation using 0–4 scale by^25^ (0 = immune = no uredinia or other macroscopic sign of infection,  = nearly immune = no uredinia, but hypersensitive necrotic or chlorotic flecks present, 1 = highly resistant = small uredinia surrounded by necrosis, 2 = moderately resistant = small to medium uredinia surrounded by chlorosis or necrosis, green island may be surrounded by chlorotic or necrotic border, 3 = moderately susceptible = medium-sized uredinia that may be associated with chlorosis, 3+, 4 = susceptible = large uredinia without chlorosis). When the susceptible checks showed the highest infection score of 33+ or 4, the inoculations were considered successful.

### Field evaluation

The germplasm was evaluated for three years (2018–2021) at PAU, Ludhiana, and at RRS Gurdaspur by following the standard protocol and relevant guidelines. Wheat germplasm lines were sown at both locations during the 2nd week of November, in a 1-m long pair rows of each genotype at a row-to-row distance of 20–22 cm. To ensure the uniform spread of rust inoculum, susceptible varieties like Agra Local, HD2967, A-9-30-1 and PBW343 were planted after every 20 rows of the test material, and infector rows were sown on the periphery of the experimental material. For comparison purposes and gene postulation, along with genotypes, NILs carrying known genes for stripe rust, susceptible and resistant checks were also sown in the field (Table 2). The stripe rust epidemic was created under field conditions by repeated spray inoculations of experimental material with uredospores of *Puccinia* *striiformis* fsp *tritici* (*Pst*)*.* Infected leaves of stripe rust susceptible varieties PBW343, Agra local, A-9-30-1, and HD2967 were immersed in water for extracting uredospores. The inoculum was prepared by suspending rust uredospores in 10 l of water using a few drops of Tween-20. The spray inoculations were done in the evening with an ultralow volume sprayer on alternate days beginning from the end of December till stripe rust appeared on the susceptible checks. Stripe rust was monitored regularly at weekly intervals starting from the second week of January to the first week of March using the modified Cobb’s Scale^26^. The host responses were graded: S = susceptible, large uredia surrounded by necrotic tissues; MS = moderately susceptible, small uredia surrounded by necrotic tissues; MR = moderately resistant, small uredia surrounded by necrotic tissues; M = Moderately resistant to moderately susceptible, small to medium sized pustules surrounded by necrosis and chlorosis; R = resistant, very small uredia surrounded by necrotic tissues^27^. The area under disease progress curve (AUDPC), and coefficient of infection (CI) were calculated by using the formula’s given below.

$${\text{AUDPC }} = \sum\limits_{n = 1}^{\infty } {\left( {\frac{{X_{i} + X_{i + 1} \left( x \right)}}{2}} \right)} \, (a_{i + 1} - t_{i} )$$

where, X_*i*_ is the rust intensity on date *i,* t_*i*_ is the time in days between *i* and date *i* + 1 and n is the number of dates on which disease was recorded. Coefficient of infection (CI) was calculated by multiplying disease severity (DS) and constant values of infection type (IT). The constant values for infection types were used based on immune = 0, R = 0.2, MR = 0.4, M = 0.6, MS = 0.8, S = 1^28^.

**Table 2:** List of near isogenic lines (NILs) in AVOCET background and Indian set of differentials for stripe rust.

S. no.	NIL	Genes	S. no.	Cross	Genes
1	MOROCCO	Null	27	*Yr35* 98M71	*Yr35*
2	AVOCET-YRA	*YRA*	28	*Yr37*	*Yr37*
3	AVOCET + YRA	*YRA*	29	CHUAN NONG 19	–
4	*YR1*/6*AOC	*Yr1*	30	*Yr51*	*Yr51*
5	SIETE CERROS T66	*Yr2*	31	KOELZ W 11192:AE	*Yr52*
6	TATARA	*Yr3*, *Yr9*, *Yr27*	32	AOC-YR/QUAIU #3	*–*
7	*YR5*/6*AOC	*Yr5*	33	QUAIU #3	*Yr54*
8	*YR6*/6*AOC	*Yr6*	34	*Yr57*	*Yr57*
9	*YR7*/6*AOC	*Yr7*	35	IRAGI	*Yr59*
10	*YR8*/6*AOC	*Yr8*	36	AOC-YR*3//LALBMONO1*4/PVN	*Yr60*
11	*YR9*/6*AOC	*Yr9*	37	*YrKK*	*YrKK*
12	*YR10*/6*AOC	*Yr10*	38	*YrAld*	*YrAld*
13	*YR15*/6*AOC	*Yr15*	39	*Yr4BL*	*Yr4BL*
14	*YR17*/6*AOC	*Yr17*	40	M10 (MUTATED C-306)/AOC-YR	–
15	*YR18*/3*AOC	*Yr18*	41	SUJATA	*Yr46*
16	*YR24*/3*AOC	*Yr24*	42	PAVON F 76	*Yr6*, *Yr7*, *Yr29*, *Yr30*, +
17	*YR26*/3*AOC	*Yr26*	43	SERI M 82	*Yr2*, *Yr9*, *Yr29*, *Yr30*, +
18	*YR27*/6*AOC	*Yr27*	44	OPATA M 85	*Yr27* +
19	AOC-YR*3/3/ALTAR 84/AE.SQ//OPATA	*Yr28*	45	SUPER KAUZ	*Yr9*, *Yr27*, *Yr18*, *Yr30*, +
20	AOC-YR*3//LALBMONO1*4/PVN	*Yr6*, *Yr7*, *Yr29* *Yr30*	46	PBW343	*Yr9*, *Yr27*, *Yr29*, +
21	AOC-YR*3/PASTOR	*Yr2*, *Yr7*, *Yr9*, *Yr29*	47	FRANCOLIN #1	*YrF,* *Yr29*
22	PASTOR	*Yr31* and *Yr29*	48	OPATA/PASTOR	*Yr27,* *Yr31*
23	*YRSP*/6*AOC	*YrSp*	49	ORIZABA	–
24	*YRCV*/6*AOC	*Yr32*	50	POLLMER	*YrPollmer*
25	*YR33*	*Yr33*	51	BICENTENARIO TCL2010	
26	*YR34*	*Yr34*	52	REBECA F2000	*Yr30,* *Yr31*

	Indian differentials set				
	Set-A	Gene	Set-B	Gene	
1	Chinese 166	*Yr1*	Hybrid 46	*Yr4*	
2	Lee	*Yr7*	Heines VII	*Yr2*+	
3	Heines Kolben	*Yr6*	Compair	*Yr8*	
4	Vilmorin 23	*Yr3*	*T.* *spelta* *album*	*Yr5*	
5	Moro	*Yr10*	Tc*6/*Lr26*	*Yr9*	
6	Strubes Dickopf	*Yrsd*	Sonalika	*Yr2*+	
7	Suwon92 X Omar	*YrSu*	Kalyansona	*Yr2*(KS)	
8	Riebesel 47/51	*Yr9*+	*Yr24*/3*AvS	*Yr24*	
9	Cappelle-Desprez	*Yr16*	*Yr15*/6*AvS	*Yr15*	
10	Carsten V	*Yr32*	*YrSP*/6*AvS	*YrSp*	

### Identification of stripe rust resistance genes by using tagged molecular markers

Under North Indian conditions against the prevalent pathotype of the stripe rust pathogen five genes namely *Yr5,* *Yr10,* *Yr15,* *Yr24,* and *Yr26* are highly effective so the tagged markers for these genes were used to identify the presence or absence of these genes in the tested germplasm. In total thirteen markers (SSR, EST-SSR, and STS) were used to profile the wheat genotypes. The Graingenes database (http://wheat.pw.usda.gov/) was employed to retrieve the sequences of available markers (Table 3) along with their previously determined chromosomal positions. Integrated DNA Technologies, Inc., synthesized the primers.

**Table 3:** *Yr* gene(s) tagged markers used to identify the stripe rust resistance genes in the test germplasm.

Gene	Marker name	Type of marker	Primer sequence	Annealing temperature (ºC)	Distance (cM)	Product size (bp)	References
*Yr5*	*Wmc175*	SSR	GATAAAATCATTATTGGGTGTCCTTT TTCAAATAATCTTTCATCAGTCAAATG	61	1.4	253 ( +)	^35^
	STS7/8	STS	GTACAATTCACCTAGAGT GCAAGTTTTCTCCCTATT	45	0.3	500 ( +)	^35^
	*Gwm120*	SSR	GATCCACCTTCCTCTCTCTC GATTATACTGGTGCCGAAAC GCAGACCTGTGTCATTGGTC GATATAGTGGCAGCAGGATACG	60	11.0	156 ( +)	^67^
*Yr10*	*Xpsp3000*	SSR		55	1.5	240 (–), 260 ( +)	^27^
	*E1*	EST-SSR	CTTGCTGGCGACCTGCTTA TGTTTCGCTCCACGCTGACT	55		754	^70^
*Yr15*	*Xgwm413*	SSR	TTTTTGGCTTATTAGACTGACTT TTGCCATAAAATACAAAATCC	60	2.5	95 ( +), 100 (–)	^67^
	*Xgwm11*	SSR	AAAAGGAACCTCAAGTGACA	50	2.1	212	^68^
			GAAAATGAGGGAGTGAGATG				
	*Xgwm273*	SSR	ATTGGACGGACAGATGCTTT AGCAGTGAGGAAGGGGAT C	55	2.1	156 ( +), 165 170, 180	^36^
	*Xbarc8*	SSR	GCGGGAATCATGCATAGGAAAACAGAA GCGGGGGCGAAACATACACATAAAAACA	50	4.2	60 ( +), 280	^35^
*Yr24/26*	*Barc181*	SSR	CGCTGGAGGGGGTAAGTCATCAC CGCAAATCAAGAACACGGGAGAAAGAA	58	6.7	180 ( +), 220 (–)	^37^
	*Barc187*	SSR	GTGGTATTTCAGGTGGAGTTGTTTTA CGGAGGAGCAGTAAGGAAGG	57	2.3	200 ( +), 220 (–), 225	^37^
	*CON-6* *(EST)*	EST-SSR	GCCGATGGGGAACTGAAT GTTGAACCGCTTGAACACC	52	0.08	Not amplified	^69^
	*Xgwm498*	SSR	GGTGGTATGGACTATGGACACT TTTGCATGGAGGCACATACT	60	1.6	160 ( +)	^66^

*cM* centimorgan, *bp* base pair.

### DNA extraction

Genomic DNA was extracted from 100 mg of fresh leaves collected from individual lines using the modified CTAB extraction method^29–31^. To remove the contaminant RNA from the DNA extracted from fresh leaves, 10 μl/ml RNase was added and then incubated at 37 ºC for 45 min.

### PCR amplification and electrophoretic separation of PCR products

After quality and quantity check the amplification using Polymerase Chain Reaction (PCR) was performed in an Eppendorf Master cycler to study the genetic polymorphism among the genotypes carrying known *Yr* genes. PCR analysis was carried out in the reaction volume of 11 μl containing the 3 μl template genomic DNA, 1.0 μl forward and reverse primers, 5 μl of 10× PCR buffer and 0.5 μl of BSA and PVP. Specific PCR amplification protocols were followed for each primer linked to different *Yr* genes. Protocol for *Yr5* markers (*Wmc175* and *Xgwm120*) was given by^32,33^, *Yr10* (*Xpsp3000*)^34^, *Yr15* (*Xgwm413* and *Xgwm273*)^35,36^, *Yr24/26* (*Barc181*)^37^ and *Barc187*^38^. PCR products were separated through 6% PAGE using PAGE apparatus (Mega-Gel System) of CBS, Scientific, USA (C-DASG-400-50) and visualized under UV light in gel documentation (SYNGENE, G: Box, USA).

## Results

### Seedling reaction test

Disease data in terms of infection types and host response were recorded on selected wheat genotypes and four susceptible checks (PBW343, HD2967, A-9-30-1 and Agra Local) at the seedling stage, as shown in Table 4. Fourteen genotypes were found to be immune (ITs 0), accounting for 29.2% of all genotypes. 28 genotypes (58.3%) were resistant (ITs1), 3 genotypes (6.3%) were moderately resistant (ITs 2), and 4 genotypes (HD2967, Agra local, PBW 343& A-9-30-1) were susceptible (ITs 4) (Table 4). The Near isogenic lines for *Yr5,* *Yr10,* *Yr15,* *Yr24/26* all showed nearly immune (0) to resistant (1) reaction.

**Table 4:** Data on infection types (ITs) at seedling stage in the selected wheat accessions against all the tested pathotypes of *Puccinia* *striiformis* f. sp. *tritici.*

IT	Accession no.
0	IC415825, IC534662, EC339610, EC664198, EC693621, EC635701, EC693322, EC635577, IC0078981-B, EC0635709, EC0610955, IC0078959-A, IC47034, IC445516,
1	EC0612504, IC530078, IC443681, IC111691, IC535470, IC336645, EC636264, EC662236, EC692262, ECO635685, EC0635659, IC0591082, IC0594933, ICO598571, EC0635701, IC0078729-A, EC0582305, EC0612509, EC0612506, IC0591055, EC0610929, EC0105966, EC0610944, IC564090, IC469420, IC529052, IC529094, IC530087
2	EC217835, EC635750, IC553914
4	PBW343, Agra Local, HD2967, A-9–30-1

### Field evaluation

Data on adult-plant stage were collected in the field based on disease severity, host response, and AUDPC (Table 5). On pooling data collected at both locations over the three crop seasons it was found that 19 lines (30.6% of total genotypes) showed no disease i.e. final rust severity (FRS) = zero and 0 AUDPC, 19 genotype lines (30.6%) showed resistance with FRS = TR-5MR (AUDPC = 100); and 7 genotype lines (14.6%) expressed a moderately resistant response with 5MS-10S final rust severity (AUDPC = 101–200). Susceptible checks; HD2967, Agra local, PBW 343, A-9-30-1) expressed 80% disease severity (80S; AUDPC = 800). NILs carrying *Yr5,* *Yr10,* *Yr15,* *Yr24/26* *genes* exhibited 0–5 MS final rust severity under artificial inoculated conditions in field.

**Table 5:** Reaction of selected wheat accessions against stripe rust infection under field condition.

FRS	Genotypes
0	IC415825, IC534662, EC339610, EC664198, EC693621, EC636264, EC662236, EC635701, EC693322, EC635577, ECO635685, IC0078981-B, EC0635709, EC0610955, EC0635659, IC0078959-A, IC47034, IC445516, IC564090, NILs(Yr5/6*AOC, Yr10/6*AOC, Yr15/6*AOC,Yr24/6*AOC, Yr26/6*AOC)
TR-5MR	IC111691, IC535470, IC336645, EC217835, EC635750, IC0591082, IC0594933, ICO598571, EC0635701, IC0078729-A, EC0612509, EC0610929, EC0612504, EC0610944, IC530078, IC443681, IC529052, IC529094, IC530087, EC0612506
5MS-10S	EC692262, EC0582305, IC0591055, EC0105966, IC553914, IC469420
80S	PBW343, Agra Local, HD2967, A-9-30-1

### Identification of *Yr* genes

Gene postulation in selected genotypes was performed using 13 *Yr* gene-tagged markers. The presence of one, two, or more than two gene combinations was observed. Table 6 shows the total number of alleles of each microsatellite marker recorded for all genotypes tested, for the detection of known *Yr* genes (*Yr5,* *Yr10,* *Yr15,* *Yr24/* *Yr26*), with the amplified allele number being given as 0 for the absence and 1 for presence.

**Table 6:** Amplification of know *Yr* genes with tagged markers on wheat germplasm lines and their allele sizes (bp).

Sl. no.	Genotypes	*Yr5*		*Yr10*	*Yr15*		*Yr24/26*	
		253 ( +)	156 ( +)	260 ( +), 240 (−)	146 ( +) 180 (−)	95 ( +), 100 (−)	180 ( +), 200 (−)	200 ( +), 220 (−)
		*Xwmc175*	*Xgwm120*	*Xsps3000*	*Xgwm273*	*Xgwm* *413*	*Barc181*	*Barc187*
1	IC111691	0	0	1	0	0	1	1
2	IC535470	1	0	0	0	0	1	1
3	IC415825	0	0	0	0	0	0	0
4	IC534662	0	0	0	1	1	0	0
5	IC336645	0	0	0	1	1	0	0
6	EC217835	0	0	0	0	0	1	1
7	EC339610	0	0	0	0	0	1	1
8	EC664198	1	1	0	0	0	0	0
9	EC693621	1	1	0	1	1	0	0
10	EC636264	1	1	0	0	0	0	0
11	EC662236	0	0	0	0	0	0	0
12	EC635701	0	0	1	1	1	0	0
13	EC693322	1	1	0	1	1	0	1
14	EC692262	1	1	0	0	0	0	1
15	EC635577	1	0	0	0	0	0	0
16	EC635750	0	0	0	0	0	1	1
17	IC47034	1	1	1	0	0	0	0
18	IC530087	0	0	0	1	1	0	0
19	IC530078	1	1	1	0	0	0	0
20	IC443681	0	0	0	0	0	0	0
21	IC445516	0	0	1	1	1	0	0
22	IC564090	0	0	0	0	0	0	0
23	IC553914	0	0	1	0	0	0	0
24	IC469420	0	0	1	1	1	0	0
25	IC529052	1	1	0	0	0	1	1
26	IC529094	0	0	1	0	0	1	1
27	EC0582305	1	1	0	1	1	0	0
28	EC0612509	1	1	0	1	1	0	0
29	EC0612506	0	0	0	0	0	1	1
30	IC0591055	0	0	0	0	0	0	0
31	EC0610929	0	0	0	0	0	0	0
32	EC0612504	1	1	0	0	0	0	0
33	EC0105966	0	0	0	0	0	1	1
34	EC0610944	0	0	1	0	0	0	0
35	ECO635685	1	1	0	1	0	0	0
36	IC0078981-B	0	0	0	0	1	0	0
37	EC0635709	0	0	0	0	0	0	0
38	EC0610955	0	0	0	1	1	1	1
39	EC0635659	1	0	0	1	1	0	1
40	IC0591082	0	0	0	0	0	0	0
41	IC0594933	0	1	1	0	0	0	0
42	ICO598571	1	0	0	0	0	1	1
43	EC0635701	0	0	0	0	0	1	0
44	IC0078729-A	0	0	0	0	0	0	0
45	IC0078959-A	0	1	0	1	1	0	0

### Postulation of *Yr5*

**Figure 1:**
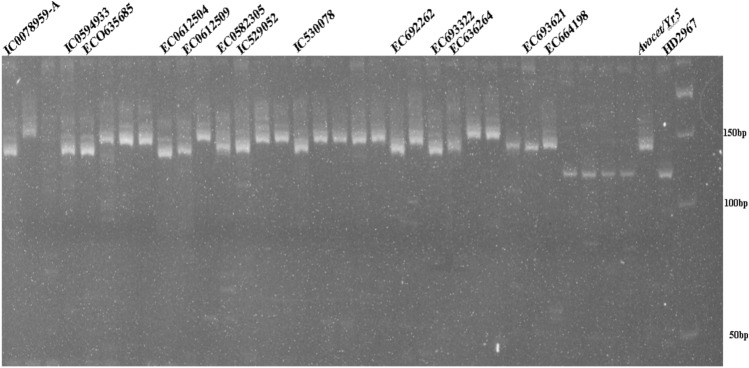
PCR amplified products of marker *Xwmc175* detecting *Yr5*gene in wheat accessions.

**Figure 2:**
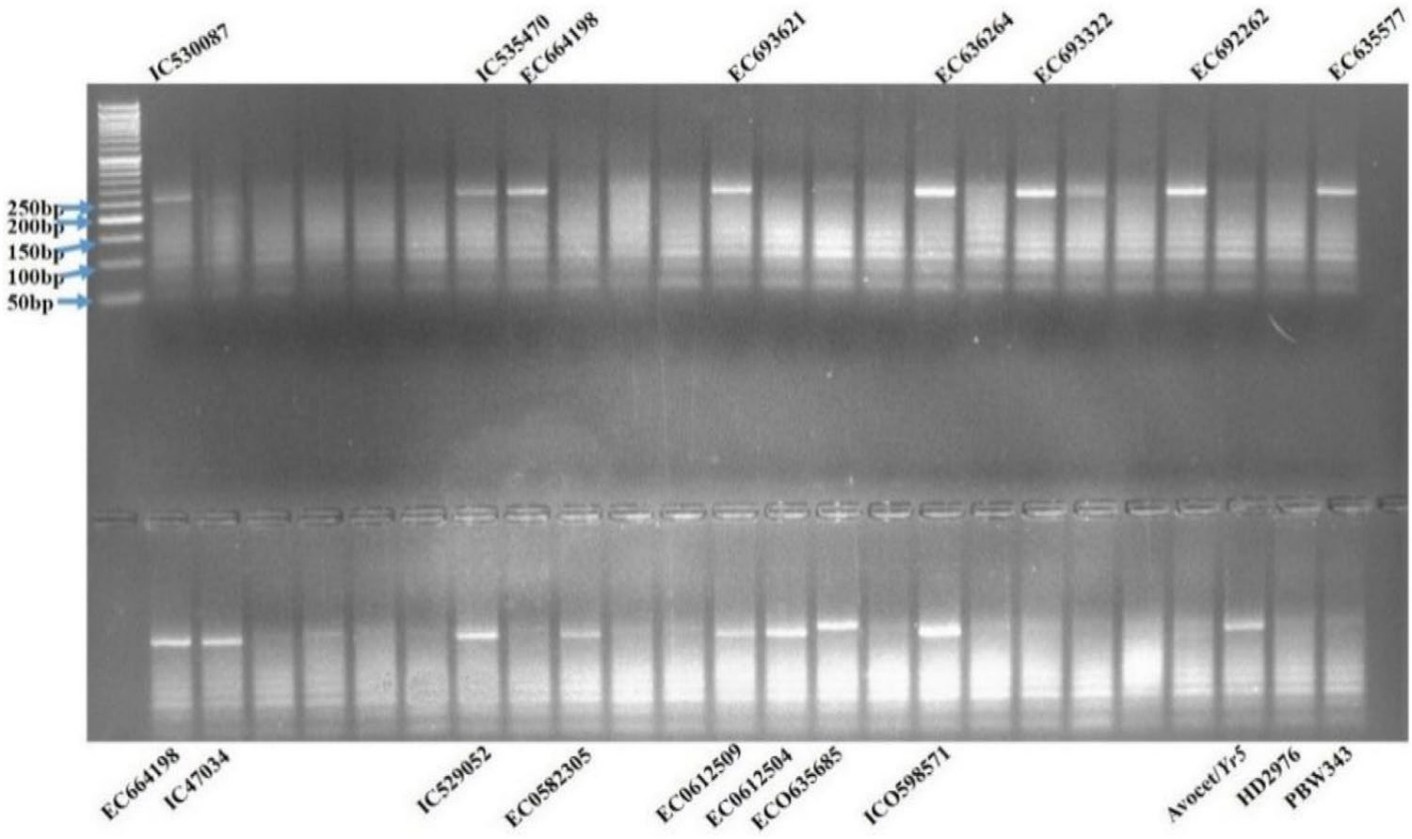
PCR amplified products of marker *Xgwm120* detecting *Yr5*gene in wheat accessions.

The dominant SSR marker *Xwmc175* is 1.4 cM away from the gene^35^. To confirm and evaluate the diagnostic potential of the markers in wheat genotypes, two microsatellite markers *Wmc175,* *Xgwm120*, and one STS marker, *STS9/10*, linked to the stripe rust resistance gene *Yr5* were used. The presence of the *Yr5* gene was associated with a product size of 253 bp in 16 lines (31.3 percent), while the other 33 wheat genotypes (68.8 percent) failed to amplify the gene (Fig. 1). Another SSR marker, *Xgwm* *120*, was found in 13 lines and is closely linked to *Yr5* amplified alleles of 156 bp (Fig. 2). Based on *Yr5* linked markers (*Wmc175* *and* *Xgwm120*), 16 genotypes (IC535470, EC693621, EC636264, EC693322, EC692262, EC635577, EC664198, IC529052, EC0582305, EC0612509, EC0612504, EC0635659, IC47034, EC0635685, IC0598571, IC0594933, IC0078959-A, EC530087, *Avocet/Yr5)* were found to have the *Yr5* gene.

### Postulation of *Yr10*

**Figure 3:**
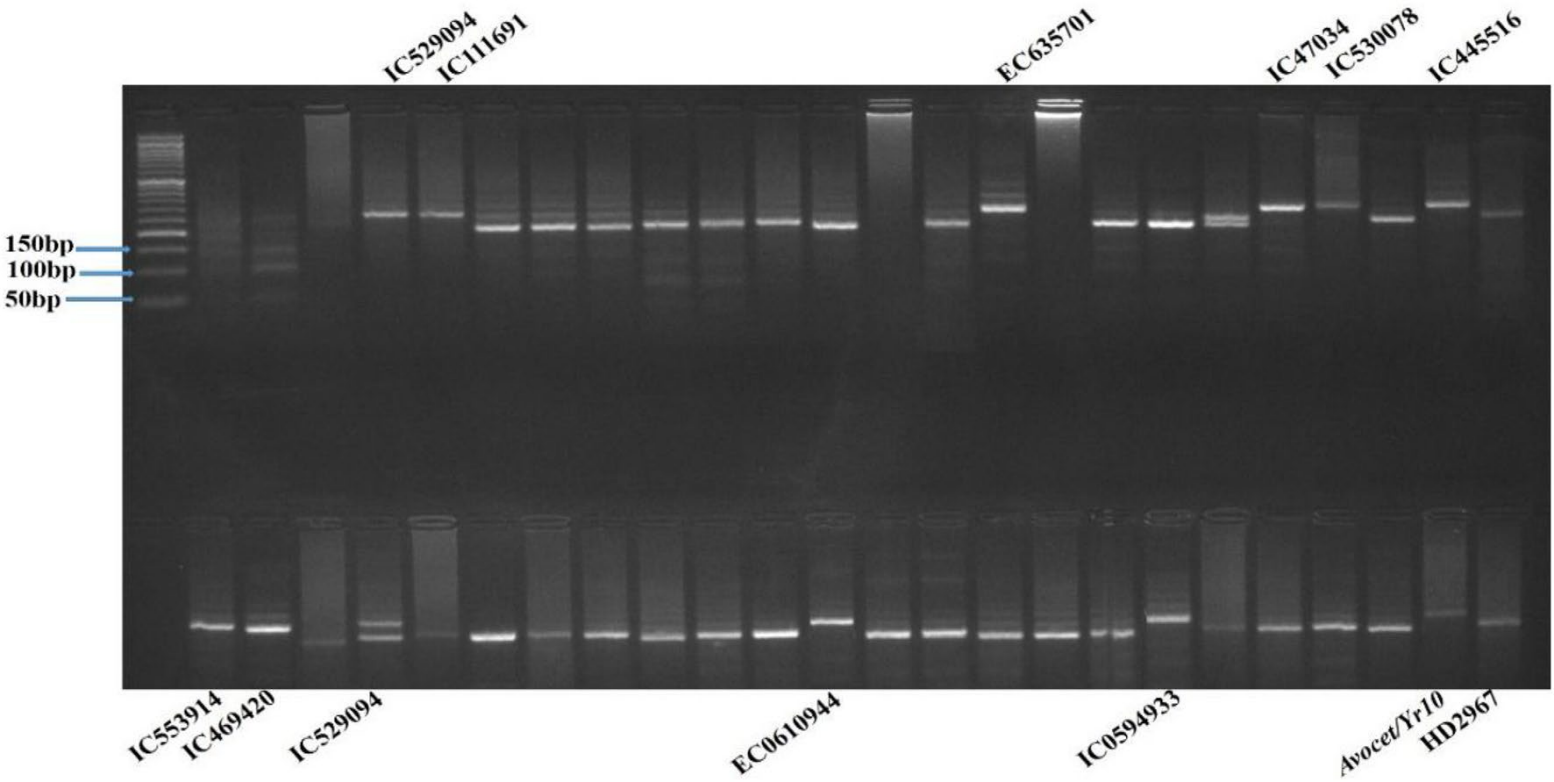
PCR amplification of *Yr10* gene using *Xpsp3000* marker, showing two types of alleles, viz*.,* 260 bp as resistant and 240 bp as susceptible.

The microsatellite marker *Xpsp3000* is co-dominantly inherited and can be used to identify genotypes of individuals at any growth stage^39^. The fragment 260 bp was amplified in ten tested entries i.e. IC529094, IC111691, EC635701, IC47034, IC530078, IC445516, IC553914, IC469420, EC0610944, and IC0594933, which shows the presence of *Yr10* gene, in rest of the 35 accessions tested it was absent (Fig. 3). In addition to the SSR *Xpsp3000* marker, the *E10* marker with a genetic distance of 0.5 cM from *Yr10*^40^ was used for detecting *Yr10* gene in test accessions but that did not work.

### Postulation of *Yr15*

**Figure 4:**
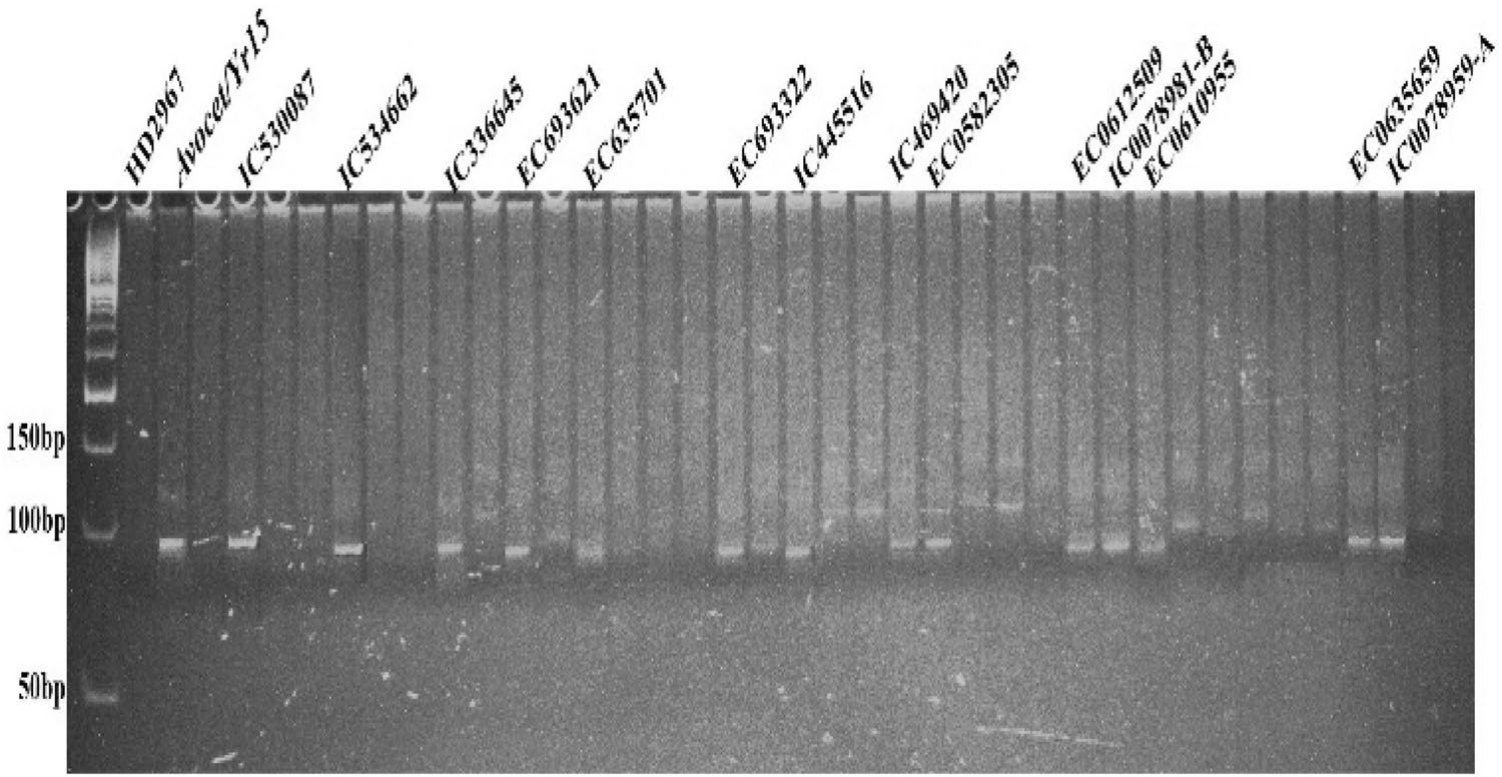
PCR amplified products of marker *Xgwm413* detecting *Yr15* gene in wheat germplasm.

**Figure 5:**
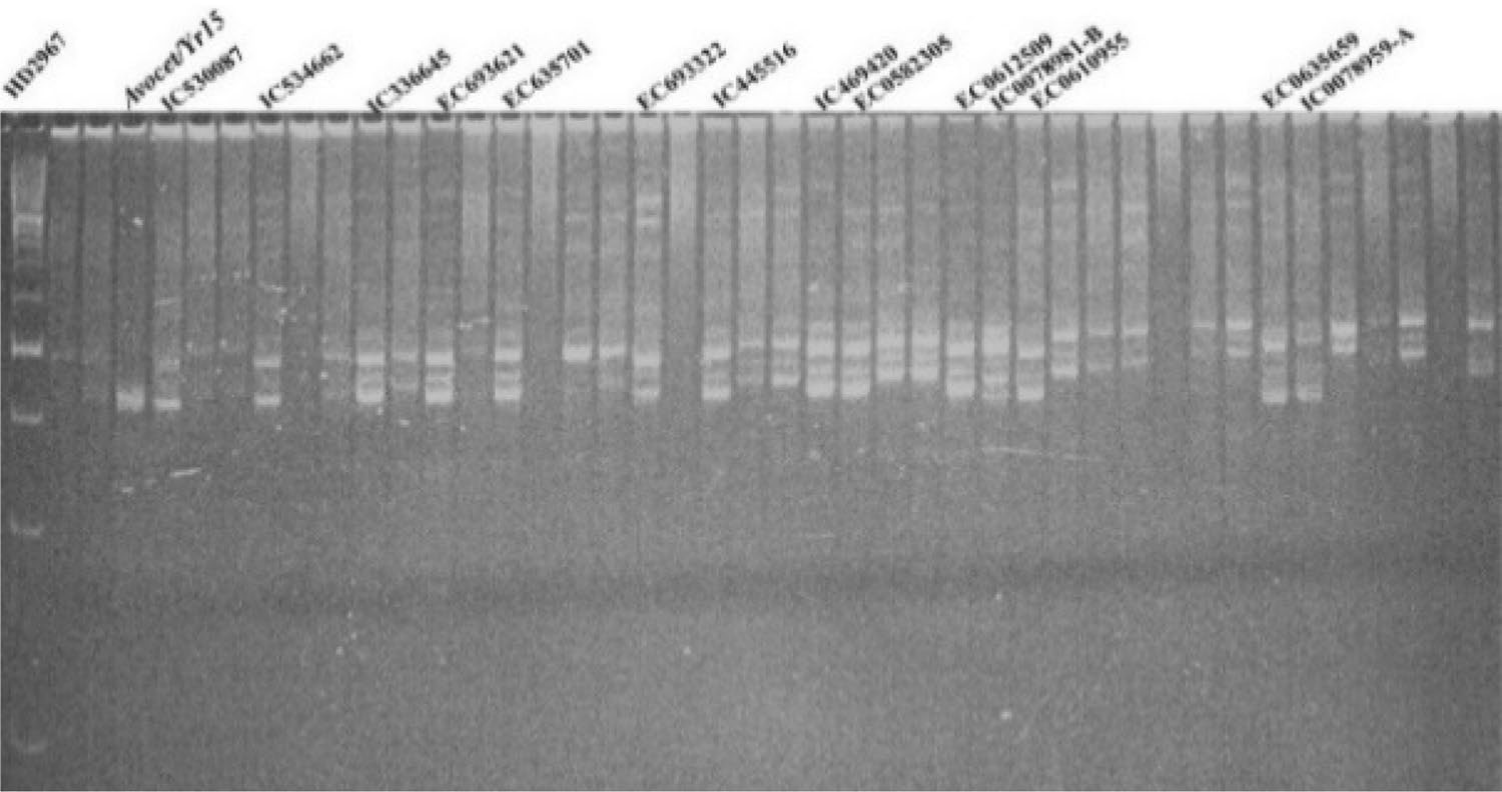
PCR amplified products of marker *Xgwm273* detecting *Yr15* gene in wheat germplasm.

*Yr15* gene diagnostics markers have been identified as *Xbarc8*, *Xgwm273*, and *Xgwm413*. These three markers were used in the current study to detect the presence of *Yr15* gene in wheat genotypes under study. The 3.5 cM proximal SSR locus *Xgwm413* produced three types of alleles (90 bp, 95 bp, and 100 bp) among the wheat genotypes used in this study. The allelic profile of *Xgwm413* showed variation. Fourteen genotypes amplified specific alleles of 90 bp, 18 genotypes amplified alleles of 95 bp, and 16 genotypes did not amplify any allele. (Fig. 4). The *Xgwm273* marker is located 2.1 cM from *Yr15* and amplified 5 different types of alleles (156 bp, 165 bp, 180, 200 bp, and 220 bp). 14 genotypes (29.2%) produced 156 bp bands specific for *Yr15* (Fig. 5). Based on confirmation of *Yr15* with both *Xgwm413* and *Xgwm273* markers, fifteen genotypes (31.3%) (IC534662, IC336645, IC530087, EC693621, EC635701, EC693322, IC445516, IC469420, EC0582305, EC0612509, IC0078981-B, EC0610955, EC0635659, IC0591082, IC0078959-A, Avocet/*Yr15*) showed the presence of *Yr15* gene.

### Postulation of *Yr24/26*

**Figure 6:**
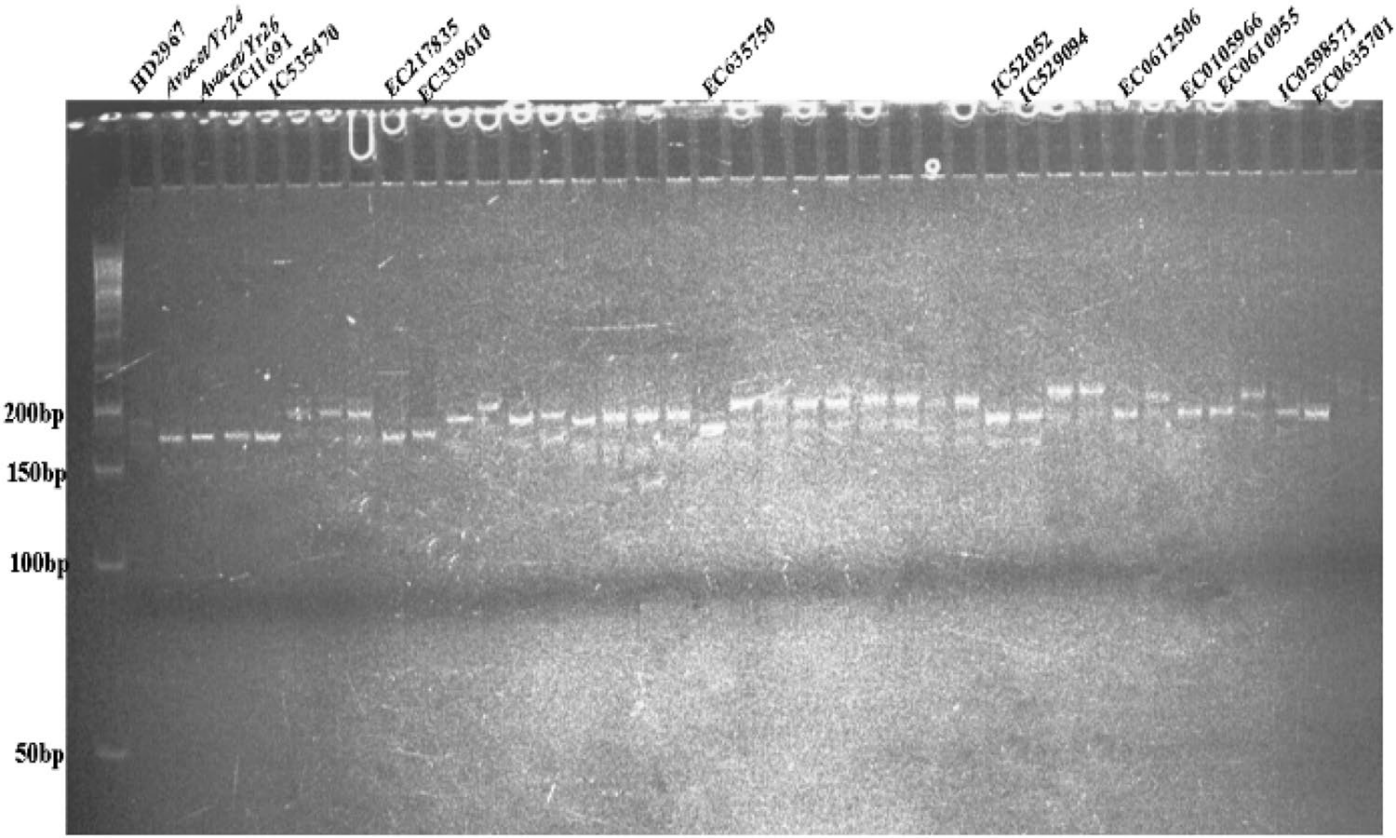
PCR amplified products of marker *Barc181* detecting *Yr24/26* gene in wheat germplasm.

**Figure 7:**
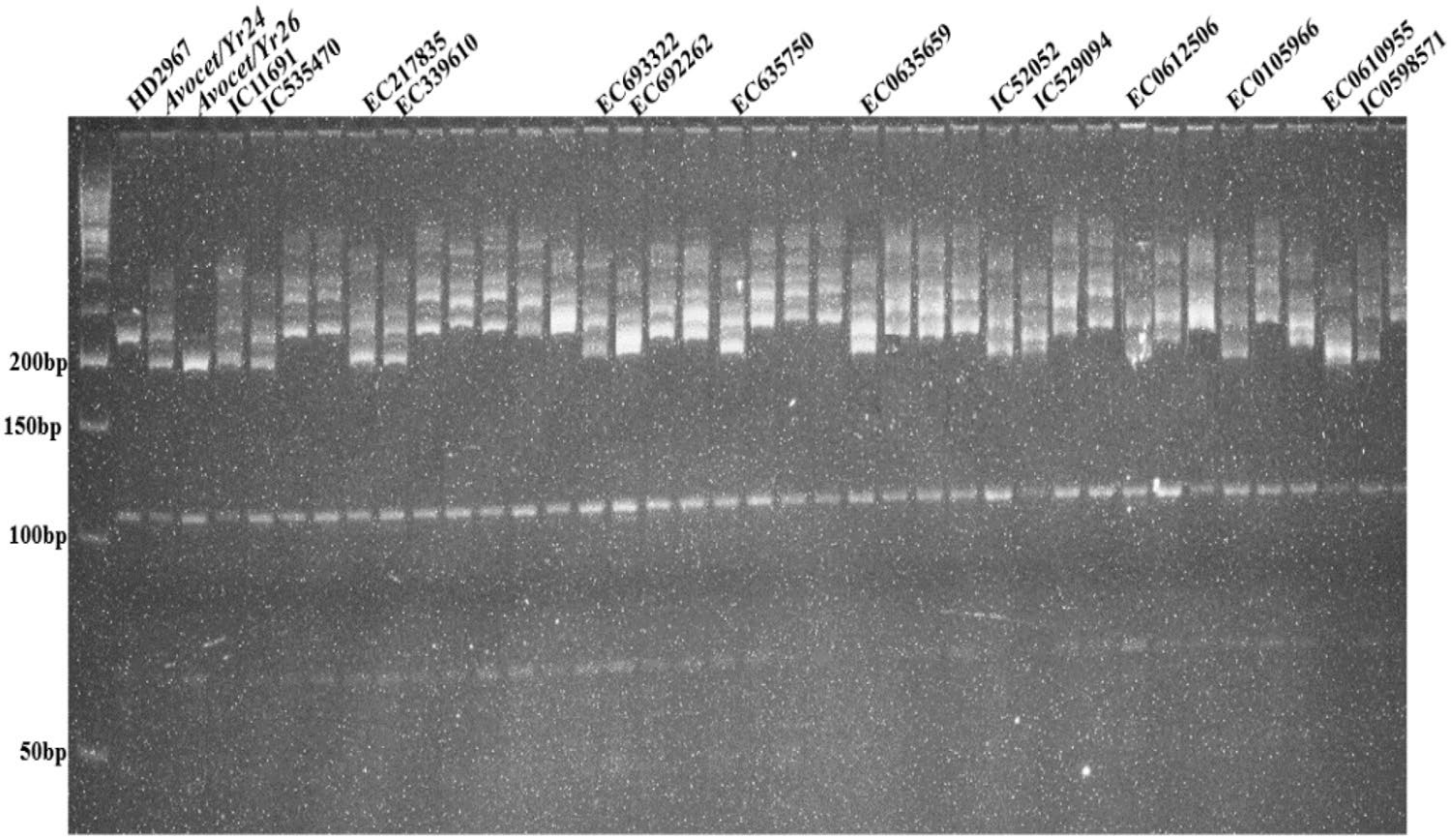
PCR amplified products of marker *Barc187* detecting *Yr24/26* gene in wheat germplasm.

The presence/absence of *Yr24/Yr26* genes was detected using two markers, *Barc181* and *Barc187*. Individual results for SSR marker *Barc181* linked with *Yr24* at 6.7 cM^37^ amplified two types of alleles; 180 bp (presence of *Yr24*) and 200 bp (absence of *Yr24*), respectively. In twelve genotypes, IC111691, IC53547, EC217835, EC33961, EC635750, IC529052, IC529094, EC0612506, EC0105966, EC0610955, ICO598571, and EC0635701 the presence of *Yr24* gene was observed as the amplified products showed the presence of 180 bp specific allele, indicating the presence of *Yr24*. SSR marker *Xbarc187*, which is 2.3 cM away from *Yr26*^37^, produces three types of alleles, 200 bp, 225 bp, and 240 bp. Fourteen genotypes i.e. IC111691, IC53547, EC217835, EC33961, EC635750, IC529052, IC529094, EC0612506, EC0105966, EC0610955, ICO598571, EC693322, EC692262 and EC0635659 were detected with the *Yr26* gene due to presence of 200 bp specific allele. Combined results with both the markers exhibited the presence of *Yr24/Yr26* in 11 genotypes (22.9%) (Figs. 6, 7).

**Figure 8:**
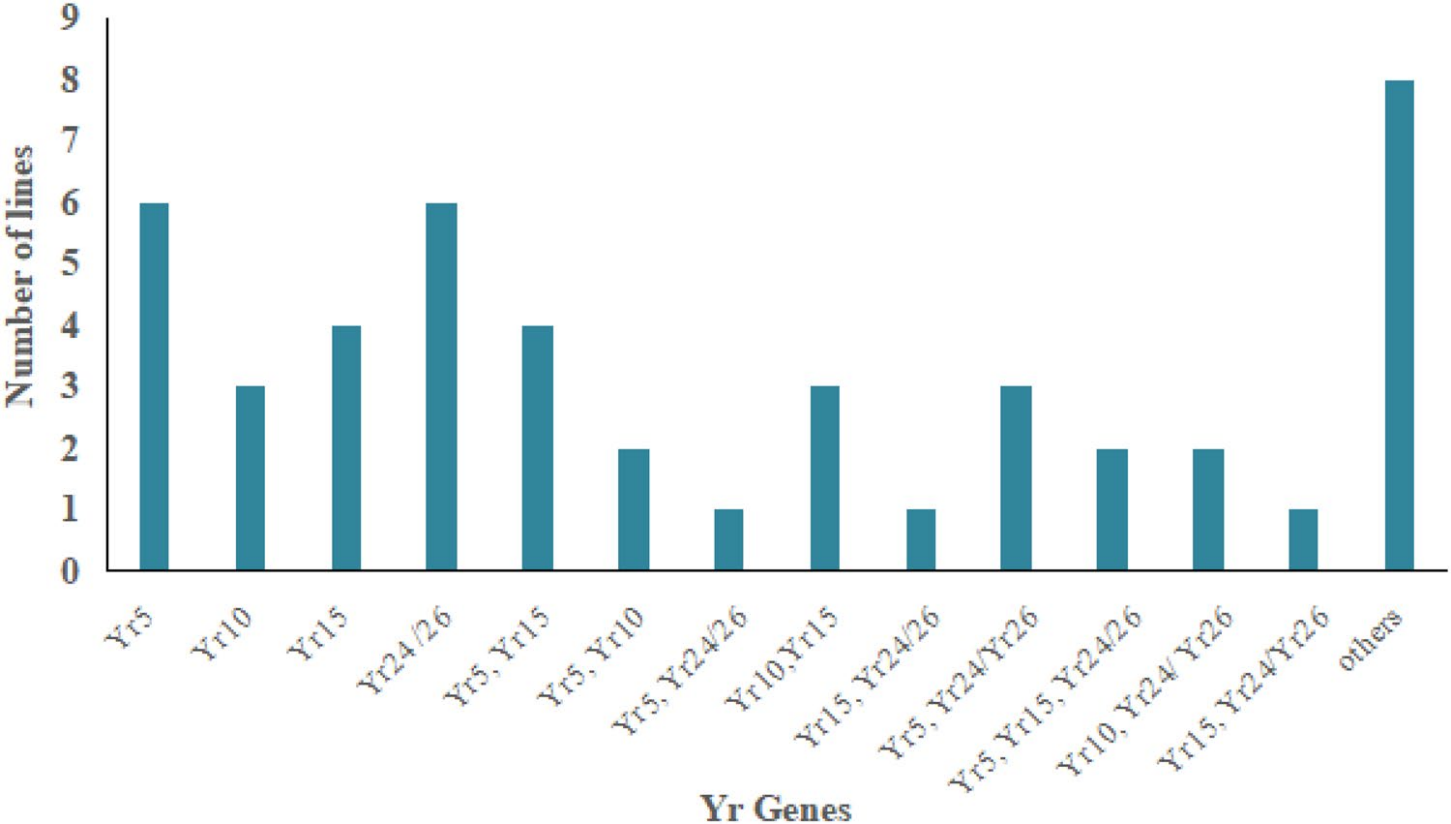
Distribution of five *Yr* resistance genes among wheat germplasm.

The distribution of these five *Yr* resistance genes among 45 wheat accessions is illustrated in Fig. 8 and Table 7. Fourteen lines were found to carry a single gene, 16 lines showed the presence of two gene combinations, and seven accessions were found to have a combination of three genes. These 45 accessions exhibited strong resistance at seedling and adult plant stage.

**Table 7:** Seedling response, AUDPC and *Yr* gene(s) postulated in wheat germplasm.

S. no.	Accession no.	Infection type			FRS	AUDPC	Postulated genes based on differential’s reaction	Results of MAS
		238S119	110S119	46S119				
1	EC664198	0	0	0	0	0	*Yr5,* *Yr10,* *Yr15,* *Yr24,* *Yr26*	*Yr5*
2	EC636264	1	1	1	0	78	*Yr5,* *Yr10,* *Yr15,* *Yr24,* *Yr26*	*Yr5*
3	EC635577	0	0	0	0	0	*Yr5,* *Yr10,* *Yr15,* *Yr24,* *Yr26*	*Yr5*
4	IC530078	1	2	1	0	108	*Yr5,* *Yr10,* *Yr15,* *Yr24,* *Yr26*	*Yr5*
5	EC0612504	1	1	0	TS	65	*Yr5,* *Yr10,* *Yr15,* *Yr24,* *Yr26*	*Yr5*
6	ECO635685	1	1	1	0	25	*Yr5,* *Yr10,* *Yr15,* *Yr24,* *Yr26*	*Yr5*
7	IC553914	2	1	2	20	183	*Yr5,* *Yr10,* *Yr15,* *Yr24,* *Yr26*	*Yr10*
8	IC530087	1	1	0	0	100	*Yr5,* *Yr10,* *Yr15,* *Yr24,* *Yr26*	*Yr10*
9	EC0610944	1	0	2	0	100	*Yr5,* *Yr10,* *Yr15,* *Yr24,* *Yr26*	*Yr10*
10	IC534662	0	0	0	0	0	*Yr5,* *Yr10,* *Yr15,* *Yr24,* *Yr26*	*Yr15*
11	IC336645	2	1	1	0	87	*Yr5,* *Yr10,* *Yr15,* *Yr24,* *Yr26*	*Yr15*
12	IC0078981-B	0	0	0	0	0	*Yr5,* *Yr10,* *Yr15,* *Yr24,* *Yr26*	*Yr15*
13	IC0591082	1	1	1	0	90	*Yr5,* *Yr10,* *Yr15,* *Yr24,* *Yr26*	*Yr15*
14	EC0635701	1	2	1	TS	52	*Yr5,* *Yr10,* *Yr15,* *Yr24,* *Yr26*	*Yr24/26*
15	EC339610	0	0	0	0	70	*Yr5,* *Yr10,* *Yr15,* *Yr24,* *Yr26*	*Yr24/26*
16	EC0612506	1	1	1	10S	52	*Yr5,* *Yr10,* *Yr15,* *Yr24,* *Yr26*	*Yr24/26*
17	EC217835	2	2	2	0	100	*Yr5,* *Yr10,* *Yr15,* *Yr24,* *Yr26*	*Yr24/26*
18	EC693621	0	0	0	0	0	*Yr5,* *Yr10,* *Yr15,* *Yr24,* *Yr26*	*Yr5,* *Yr15*
19	EC692262	1	1	1	10S	38	*Yr5,* *Yr10,* *Yr15,* *Yr24,* *Yr26*	*Yr5,* *Yr24/26*
20	EC0105966	2	10	0	10S	173	*Yr5,* *Yr10,* *Yr15,* *Yr24,* *Yr26*	*Yr24/* *Yr26*
21	EC635750	2	2	2	0	98	*Yr5,* *Yr10,* *Yr15,* *Yr24,* *Yr26*	*Yr24/* *Yr26*
22	IC0594933	1	1	1	0	35	*Yr5,* *Yr10,* *Yr15,* *Yr24,* *Yr26*	*Yr5,* *Yr10*
23	IC47034	0	0	0	0	0	*Yr5,* *Yr10,* *Yr15,* *Yr24,* *Yr26*	*Yr5,* *Yr10*
24	EC0612509	1	1	1	0	0	*Yr5,* *Yr10,* *Yr15,* *Yr24,* *Yr26*	*Yr5,* *Yr15*
25	EC635701	0	0	0	0	0	*Yr5,* *Yr10,* *Yr15,* *Yr24,* *Yr26*	*Yr10,Yr15*
26	EC0582305	0	1	1	5MR	108	*Yr5,* *Yr10,* *Yr15,* *Yr24,* *Yr26*	*Yr5,* *Yr15*
26	IC0078959-A	0	0	0	0	0	*Yr5,* *Yr10,* *Yr15,* *Yr24,* *Yr26*	*Yr5,* *Yr15*
28	IC445516	0	0	0	0	0	*Yr5,* *Yr10,* *Yr15,* *Yr24,* *Yr26*	*Yr10,* *Yr15*
29	IC469420	1	1	1	10	150	*Yr5,* *Yr10,* *Yr15,* *Yr24,* *Yr26*	*Yr10,* *Yr15*
30	EC0635659	0	1	1	0	22	*Yr5,* *Yr10,* *Yr15,* *Yr24,* *Yr26*	*Yr5,* *Yr15,* *Yr24/26*
31	IC535470	1	1	1	0	58	*Yr5,* *Yr10,* *Yr15,* *Yr24,* *Yr26*	*Yr5,* *Yr24/Yr26*
32	EC693322	0	0	0	0	0	*Yr5,* *Yr10,* *Yr15,* *Yr24,* *Yr26*	*Yr5,* *Yr15,* *Yr24/26*
33	IC529052	0	1	1	0	78	*Yr5,* *Yr10,* *Yr15,* *Yr24,* *Yr26*	*Yr5,* *Yr24/26*
34	ICO598571	1	1	1	0	59	*Yr5,* *Yr10,* *Yr15,* *Yr24,* *Yr26*	*Yr5,* *Yr24,* *Yr26*
35	IC111691	1	2	1	0	100	*Yr5,* *Yr10,* *Yr15,* *Yr24,* *Yr26*	*Yr10,* *Yr24/Yr26*
36	IC529094	0	1	1	0	75	*Yr5,* *Yr10,* *Yr15,* *Yr24,* *Yr26*	*Yr10,* *Yr24/Yr26*
37	EC0610955	0	0	0	0	0	*Yr5,* *Yr10,* *Yr15,* *Yr24,* *Yr26*	*Yr15,* *Yr24/26*
38	IC415825	0	0	0	0	0	*Yr5,* *Yr10,* *Yr15,* *Yr24,* *Yr26*	Other than *Yr5,* *Yr10,Yr15,* *Yr24/* *Yr26*
39	EC662236	1	1	1	0	0	*Yr5,* *Yr10,* *Yr15,* *Yr24,* *Yr26*	Other than *Yr5,* *Yr10,Yr15,* *Yr24/* *Yr26*
40	IC443681	1	1	1	0	75	*Yr5,* *Yr10,* *Yr15,* *Yr24,* *Yr26*	Other than *Yr5,* *Yr10,Yr15,* *Yr24/* *Yr26*
41	IC564090	1	1	2	0	125	*Yr5,* *Yr10,* *Yr15,* *Yr24,* *Yr26*	Other than *Yr5,* *Yr10,Yr15,* *Yr24/* *Yr26*
42	IC0591055		2	1	10S	175	*Yr5,* *Yr10,* *Yr15,* *Yr24,* *Yr26*	Other than *Yr5,* *Yr10,Yr15,* *Yr24/* *Yr26*
43	EC0610929	1	1	0	TR	58	*Yr5,* *Yr10,* *Yr15,* *Yr24,* *Yr26*	Other than *Yr5,* *Yr10,Yr15,* *Yr24/* *Yr26*
44	IC0078729-A	2	1	1	0	80	*Yr5,* *Yr10,* *Yr15,* *Yr24,* *Yr26*	Other than *Yr5,* *Yr10,Yr15,* *Yr24/* *Yr26*
45	EC0635709	0	0	0	0	0	*Yr5,* *Yr10,* *Yr15,* *Yr24,* *Yr26*	Other than *Yr5,* *Yr10,Yr15,* *Yr24/* *Yr26*
46	Yr5*Avocet	1	0	0	0	0	*Positive* *control*	*Yr5*
47	Yr10*Avocet	1	1	1	0	0	*Positive* *control*	*Yr10*
48	Yr15*Avocet	1	1	0	0	0	*Positive* *control*	*Yr15*
49	Yr24*Avocet	1	1	1	0	20	*Positive* *control*	*Yr24/26*
50	Yr26*Avocet	1	1	1	0	20	*Positive* *control*	*Yr24/26*

## Discussion

Many sources of wheat yellow rust genetic resistance have proven ineffective after only a short period of deployment^41^. Monoculture of wheat cultivar PBW343 in Punjab, India resulted in the emergence of a new *Pst* race, 78S84, and a decrease in the predominance of *Pst* race 46S119. Because the new race 78S84 was virulent on *Yr27* thus PBW343 became vulnerable. During 2008–2010, the race 78S84 was found in more than 80% of infected samples collected from North Western Plains Zone of India. However, as PBW343 was replaced by cultivars with R genes other than *Yr27*, 46S119 became prevalent once more, while 78S84 became the least common one (5%). Three new races, 238S119, 110S119 and 46S117, have also emerged in Punjab and their prevalence’s are increasing with every passing year^42^. The knockdown of race-specific single genes is always a concern for plant breeders. The pyramiding of more than one R gene into a single cultivar by MAS is the best way to develop cultivars with durable resistance and slows down the process of pathogen evolution. against current prevalent and new emerging *Pst* races^43^. In fact, continuous search for novel source of donor genotype is warranted for identification of resistance genes and their deployment in new cultivars or cultivars with defeated genes and unexploited genebank material is the best option for such kind of study. Forty-five genotypes including indigenous (IC-) and exotic collections (EC-) were selected out of ~ 1500 diverse set of genebank germplasm accessions screened for yellow rust disease during 2014–15 and 2015–16. Subsequently, this study was conducted to postulate the *Yr* genes in selected and pre-screened germplasm accessions for yellow rust resistance. Therefore, this study was conducted to postulate the *Yr* genes in selected genotypes (45) using *Yr* gene-tagged markers (13) and to assess the contribution of *Yr* genes to the current status of *Pst* resistance. In the current study, we have found three types (single, two, and three genes) combinations among the germplasm under study. Fourteen lines of wheat germplasm were found to carry a single gene, 16 lines showed the presence of two gene combinations, and seven genotypes were found to have a combination of three genes. These 45 genotypes lines exhibited strong resistance at seedling as well as at adult plant stage. Out of 45 germplasm lines, eight germplasm lines showed no amplification with any of the markers used, which means some other already known gene (s) or some new gene (s) may be present which confers a good level of resistance. In the rest of the 38 lines, the amplification was achieved with one/two/more of the tagged *Yr* gene markers, indicating the presence of single or multiple genes. Rani et al.^44^ found two genotypes (VL 3002 and VL 1009) with 15 *Yr* genes, followed by 14 genes in HI8759 and VL3010. Sobia et al.^43^ and Yuan et al.^45^ studied the frequency of *Yr* genes among wheat genotypes by using tagged markers. Zheng et al.^46^ also did the molecular characterization of 330 leading wheat cultivars and 164 advanced breeding lines in China and identified *Yr9,* *Yr17,* *Yr18*, and *Yr26* in 134 (29.4%), 45 (9.1%), 10 (2%) and 15 (3%) entries, respectively. Yan et al. ^47^ observed *Yr* *1*, *Yr* *13*, *Yr* *18*, *Yr* *14a*, *Yr* *26*, *Yr* *34* and *Yr* *46* either singly or in combination in twenty-five cultivars by testing them in the field during 2014–2018 crop seasons against stripe rust and also by using eleven molecular markers associated with known *Yr* genes. *Yr5* has been shown to be effective against all rust virulent races in North America ^6,48,49^, China, Iran ^6,50^, Turkey, and India ^51^. The stripe rust resistance gene *Yr5*, which was derived from *Triticum* *spelta* var *album*, is a race-specific R-gene effective at both seedling and adult plant growth stages and is located on chromosome 2BL ^52^. In our study *Yr5* presence was detected in eighteen (40%) lines with two linked markers, i.e., *Xwmc175* and *Xgwm120.* Naruoka et al. ^32^ found six lines out of 13 carrying lines carrying the resistance-linked allele using the Xwmc175 marker. *STS-9/10*, developed by Chen et al. ^49^, co-segregates with the *Yr5* locus and amplified fragments of 439 or 433 bp for resistant or susceptible plants, respectively. Using the *S19M93* molecular marker, Ullah et al.^53^ discovered an 89% polymorphism rate of the *Yr5* gene in 99 Pakistan wheat lines. The *Yr5* gene was not amplified in any of the 45 genotypes using STS9/10. In India, to date, the *Yr5* gene is effective against all prevalent races and can be an effective source for stripe rust resistance when used singly or in combination with other resistant genes. The *Yr10* gene was also found effective against all the *Pst* races prevalent in India, Iran, China, Pakistan, and the United States^54^. The marker *Xpsp3000*, located at the end of chromosome 1BS, is 1.2 cM away from the stripe rust-resistant gene, *Yr10* ^55^ which is co-dominantly inherited and can be used to identify genotypes of individuals at any growth stage^56^ and is linked with brown glume color (0.2 cM) in wheat genotypes PI 178,383 and Moro^57^, while *T.* *spleta* 415 and *T.* *vavilovii* AUS22498 have white glumes^34,58^. Resistant genotypes with brown glumes during the phenotypic evaluation were observed in nine genotypes, which was further confirmed with the tagged marker *Xpsp3000* yielded a specific allele of 260 bp. Bariana et al.^34^ described two *Yr10* alleles, *Yr10* and *Yrvav,* and stated *Yr10-*containing varieties amplified 258-260bps fragments, whereas *Yrvav-*containing varieties amplified 285 bp and 240 bp for varieties lacking the *Yr10* gene. Similarly, *Yr15* is currently the most common all-stage resistance gene used in breeding programs it is effective against all identified races in the United States^59^. *Yr15*, a dominant gene derived from *Triticum* *dicoccoides*, is found on chromosome 1BS ^60^. Sun et al. ^61^, Peng et al.^62^, and Murphy et al. ^35^ mapped the *Yr15* gene to a 6.4 cM interval flanked by markers *Xbarc8,* located 3.9 cM to the distal side*,* *Xgwm413* located 2.5 cM to the proximal side, and *Xgwm273* located at 2.5 cM and 2.1 cM to the proximal side. On the basis of confirmation with two linked markers, i.e., *Xgwm413* and *Xgwm273*, *Yr15* was detected in fourteen lines accounting for (31%) of the total genotypes. Kokhmetova et al. ^63^ used *Xbarc*8 and *Xgwm413* markers to confirm the presence of *Yr15* in seven genotypes (10%) that carried the *Yr15* gene. *Barc8* did not work well under our conditions. *Yr15* and its flanking SSR markers *Xbarc8* and *Xgwm413* are separated by 3.9 and 2.5 cM, respectively. The two markers that have been most frequently used to incorporate *Yr15* into wheat cultivars are *Xbarc8* and *Xgwm413* ^35,64^, but in our conditions, *Xbarc8* did not work. *Yr24*, discovered from the K733 accession of *T.* *turgidum* var. *durum*, confers stripe rust resistance at all stages, and the *Yr26* stripe rust resistant gene was discovered on chromosome 1B in the *T.* *turgidum* *durum* line^65^. Due to their similar infection types against stripe rust isolates, *Yr24* and *Yr26* are thought to be identical genes^66^ and further demonstrated by Clemence et al. ^39^. Combined results with both the markers exhibited the presence of *Yr24/Yr26* in 11 genotypes (22.9%), namely IC111691, IC535470, EC217835, EC339610, EC635750, IC529052, IC529094, EC0612506, EC0105966, EC0610955, and ICO598571. In EC0635701 *Yr24/26* were detected by *Barc181* whereas in EC693322, EC692262, and EC0635659 genotypes they were detected by *XBarc187.* The *Yr5,* *Yr10,* *Yr15,* *Yr24/Yr26* genes, which are specific to races, are still effective against the present dominant races prevalent in north western plains zone of India. To increase their durability, they have to be use in combination with other genes.

## Data Availability

The datasets generated during the current study will be available from the corresponding author on reasonable request.
