# Author Correction: Stripe rust resistance gene(s) postulation in wheat germplasm with the help of differentials and tagged molecular markers

**DOI:** 10.1038/s41598-023-37367-8

**Published:** 2023-06-22

**Authors:** Mohammad Waris Haider, Jaspal Kaur, Ritu Bala, Sandeep Singh, Puja Srivastava, Achla Sharma, Rohtas Singh, Jyoti Kumari

**Affiliations:** 1 Department of Plant Pathology, PAU, Ludhiana, India; 2 Department of Plant Breeding and Genetics, PAU, Ludhiana, India; 3 National Bureau of Plant Genetic Resources, New Delhi, India

Correction to: *Scientific Reports* https://doi.org/10.1038/s41598-023-36197-y, published online 02 June 2023

The original version of this Article contained an error in the name of author Mohammad Waris Haider, which was incorrectly given as Mohammad Haider Waris.

The original Article has been corrected.

